# A Real-Life Survey of Venous Thromboembolic Events Occurring in Myeloma Patients Treated in Third Line with Second-Generation Novel Agents

**DOI:** 10.3390/jcm9092876

**Published:** 2020-09-05

**Authors:** Valeria Calafiore, Silvia Giamporcaro, Concetta Conticello, Alessandra Romano, Marina Parisi, Gaetano Giuffrida, Daniele Tibullo, Francesco Di Raimondo, Salvatore Santo Signorelli

**Affiliations:** 1 Division of Hematology, AOU Policlinico, 95123 Catania, Italy; valeriacalaf@gmail.com (V.C.); ettaconticello@gmail.com (C.C.); marinaparisi@hotmail.it (M.P.); gagimapi@infinito.it (G.G.); diraimon@unict.it (F.D.R.); 2 General Medicine Unit, AOU Policlinico, Department of Clinical & Experimental Medicine, University of Catania, 95123 Catania, Italy; silvia.giamporcaro8T@gmail.com (S.G.); ssignore@unict.it (S.S.S.); 3 Division of Hematology, Department of General Surgery and Medical-Surgical Specialties, University of Catania, 95123 Catania, Italy; 4 Section of Biochemistry, Department of Biomedical and Biotechnological Sciences, University of Catania, 95123 Catania, Italy

**Keywords:** multiple myeloma, venous thromboembolic events, pomalidomide, carfilzomib

## Abstract

Compared to the general population, patients with multiple myeloma (MM) have a nine-fold increased risk of developing venous thromboembolism (VTE). Little is known about VTE prophylaxis in relapsed/refractory (RR) MM patients treated with next generation anti-myeloma drugs, such as pomalidomide (Poma) and carfilzomib (K), and monoclonal antibodies daratumumab (Dara) and elotuzumab (Elo), alone or in combination with dexamethasone at high- (D, 40 mg/week) or low-dose (d, 20 mg/week). Here, we describe the incidence of VTE in a retrospective cohort of 112 consecutive relapsed and refractory myeloma (RRMM) patients who received a third line of treatment from April 2013 to February 2020. Anti-MM regimens included combinations of pomalidomide and dexamethasone (PomaD, *N* = 61), carfilzomib, lenalidomide and dexamethasone (KRd, *N* = 31), and elotuzumab, lenalidomide and dexamethasone (EloRd, *N* = 10), while the remaining 10 patients received daratumumab as a single agent. According to National Comprehnsive Cancer Network (NCCN), International Myeloma Working Group (IMWG) and 2015 European Myeloma Network (EMN) guidelines, 42 patients (38%) were classified as high-risk patients. According to the IMPEDE VTE score, 32 patients (28%) were classified as low-risk, with a score ≤ 3 (most of them in the PomaD and Dara group), 70 (63%) were classified as intermediate-risk, with a score of 4–7 (most of them in PomaD and KRd group), and 10 (9%) were classified as high-risk, with a score ≥8 (most of them in the PomaD group). All patients received a prophylaxis, consisting generally of low-doses of acetylsalicylic acid. VTE was recorded in 9% of our patients, all of them with an intermediate or high-risk IMPEDE score, treated with low doses aspirin (ASA). No VTE occurred in patients treated with daratumumab. Thus, our real-life experience documents that (1) in RRMM patients treated with continuative regimens of third line, the incidence of VTE is similar to the setting of newly-diagnosed patients; (2) many patients in real-life received prophylaxis with ASA, irrespective of the risk classification; (3) the IMPEDE VTE score seems to be more appropriate to define the risk categories. Randomized clinical trials are required to better define the VTE prophylaxis strategy in the RRMM setting.

## 1. Introduction

The association between thrombosis and malignant disease has been recognized for many years. The risk of venous thromboembolism (VTE) varies with cancer type and has been reported to be higher in gastrointestinal, ovarian, lung cancer and hematological malignancies [1]. Multiple Myeloma (MM) is a plasma cells malignancy virtually always preceded by an asymptomatic precursor state, such as monoclonal gammopathy of undetermined significance (MGUS) [2]. Patients with MGUS and MM have an increased incidence of VTE. Indeed, the risk of thrombotic events is about three times higher than patients with MGUS, and nine times higher compared to healthy subjects [2,3,4,5]. Reasons for this elevated risk of VTE are not fully understood but it has been widely described that in MM there are changes that involve the microenvironment [6] and are associated with a prothrombotic state, characterized by high levels of inflammatory cytokines (such as TNF-a, NFkB, and IL-6) [3], upregulation of prothrombotic mediators (such as tissue factor, fibrinogen, von Willebrand Factor) and down-regulation of antithrombotic factors (such as thrombomodulin, endothelial protein C receptor, and tissue plasminogen activator) [7,8,9]. In addition, in MM an acquired activated protein C resistance [10], not related to the presence of Factor V Leiden, has been described as a transitory abnormality that could be responsible for an increased risk of venous thromboembolism [11]. Finally, high levels of monoclonal (M) component can induce hyperviscosity which leads to an increased thromboembolic risk [12].

The most common venous thrombosis observed in MM is deep vein thrombosis (DVT) in the lower extremities, followed by central venous catheter-related thrombosis, pulmonary embolism, and arm vein thrombosis.

Introduction of novel agents in the therapeutic armamentarium of MM has greatly improved response to treatment and overall survival [13], also for relapsed/refractory (RR) patients [14]. Three classes of drugs have changed the natural history of MM: proteasome inhibitors (PIs), immunomodulatory drugs (IMiDs) [14,15,16] immunotherapy and monoclonal antibodies (moAb), burdened with new or additional side effects. In particular, thrombosis occurred in about 10% of MM patients treated with chemotherapy [17], while up to 24% of patients treated with the immunomodulatory drug (IMiD) thalidomide experienced VTE complications, particularly when it is administrated in combination with other agents (dexamethasone and/or cytotoxic chemotherapy) [7,17,18,19,20]. Second generation IMiD lenalidomide may have similar effects on increasing VTE [7,18,19,21,22,23,24,25,26,27,28,29,30,31,32,33].

Because of the increased risk of development of VTE in MM, in 2008 the International Myeloma Working Group (IMWG) published recommendations for thromboprophylaxis in MM-patients treated with thalidomide or lenalidomide which consider VTE patient risk classes [34]. Factors associated with thrombosis were listed as age, history of VTE, central venous catheter, comorbidities (infections, diabetes and cardiac disease), immobilization, surgery and inherited thrombophilia. The presence of MM at diagnosis or hyperviscosity were considered additional risk factors [35]. When no or one risk factor for thromboembolism is present, patients are classified as low risk. If two or more myeloma or individual risk factors are present, or when high dose of dexamethasone, multi agent chemotherapy or doxorubicin are administered, patients are classified as high risk [34].

According to the current recommendations, low-risk patients on IMiDs plus dexamethasone should be treated with low-dose aspirin (ASA) while high-risk patients should receive prophylactic low-molecular-weight heparin (LMWH) or therapeutic dose of warfarin for 4–6 months followed by ASA treatment [32,34,36]. In case of VTE, anticoagulation therapy should be started, and anti-MM treatment should be temporarily interrupted, restarting once a stable anticoagulation is achieved [37]. These recommendations were then taken up and confirmed by the 2015 European Myeloma Network (EMN) [38] and 2017 ESMO guidelines [39]. Limited data are available about thrombotic risk in patients on treatment with pomalidomie but, as with the other IMiDs, it seems to determine an increased risk of thrombosis [17,25,40,41,42,43,44]. With regard to the second-generation proteasome inhibitor Carfilzomib, phase III trial ASPIRE, which compares the efficacy of lenalidomide-dexamethasone versus carfilzomib-lenalidomide-dexamethasone, shows a higher incidence of VTE in the three-drugs arm (13% vs. 6%), despite the mandatory use of thromboprophylaxis [45]. Little is known about vascular toxicity of Carfilzomib [46] and more studies are needed to understand the underlying mechanism of endothelial injury [46,47]. Concerning ixazomib, elotuzumab and daratumumab, VTE is not reported as an adverse event for these drugs; thus, no thromboprophylaxis is currently recommended [5,7,48]. However, recommendations on next generation myeloma drugs are derived from clinical trials where patients are selected and do not fully reflect the heterogeneity of patients in the real-life setting, in the presence of multiple risk factors for VTE [17,48,49,50].

The aim of our study was to evaluate frequency of VTE in a retrospective cohort of MM patients treated with novel anti MM agents who received VTE prophylaxis in a real-life setting.

## 2. Patients’ Selection and Methods

### 2.1. Patients’ Disposal

We performed a retrospective monocentric study, including 112 consecutive patients with relapsed and refractory myeloma (RRMM) from April 2013 to February 2020 at the Division of Hematology, University Hospital (Catania, Italy). As part of their third-line salvage regimen, all patients received second-generation drugs, outside from clinical trials, according to the evidence included in the ESMO guidelines in 2017 [39]. Patients’ characteristics are summarized in Table 1. Details and schedule of treatments are described in Appendix A. The study was approved by the local institutional review board.

Each patient’s medical history was recorded on monthly basis, on day 1 of each cycle. Physical examinations were conducted, including weight evaluation, physical examination of legs and CVC insertion if present. Blood was collected for hematologic, coagulative tests, renal and liver function tests on day 1 of each course.

All VTE were diagnosed by duplex compressive Doppler ultrasonography, performed in a single complete study of the inferior arts, of the deep veins from the inguinal ligament to the ankle (including posterior tibial and peroneal veins in the calf), right and left common femoral vein spectral Doppler waveforms (to evaluate symmetry), popliteal spectral Doppler and color Doppler images. Patients were addressed to perform the analysis in case of clinical suspicion, such as leg swollen or localized tenderness along the distribution of the deep venous system, according to recent guidelines [35]. Computed tomography (CT) and pulmonary angiography was performed as clinically indicated. For the final analysis, we included only objective VTE diagnosis.

### 2.2. Patients’ Concomitant Treatments

Concomitant medications included anti-infectious prophylaxis, consisting of Trimetoprim and Sulfametoxazole 800 mg bis in die only two days per week and Acyclovir 200–400 mg daily. Secondary prophylaxis with sub-cutaneous erythropoietin (EPO) was admitted according to ESMO guidelines [39].

### 2.3. VTE Thromboprophylaxis

VTE risk was evaluated according to IMWG recommendations for thromboprophylaxis in MM-patients [22]. Given 1 point to every risk factor, low risk patients had no or one patient, MM or therapy-related risk factors (score 0–1); in the case of two or more risk factors, patients were classified as high-risk. Antithrombotic prophylaxis consisted of ASA 100 mg per os once daily, or LMWH or other antiplatelet agents, such as clopidogrel, tyclopidine or ticagrelor. Five patients refused VTE thromboprophylaxis. No patients received prophylaxis with oral anticoagulants.

### 2.4. Statistical Analysis

Descriptive statistics was used to evaluate patients-related risk factors. Fisher exact test was used for nominal variables with two-categories to appreciate any difference due to treatment.

## 3. Results

### 3.1. Evaluation of VTE Risk Factors

**Figure 1:**
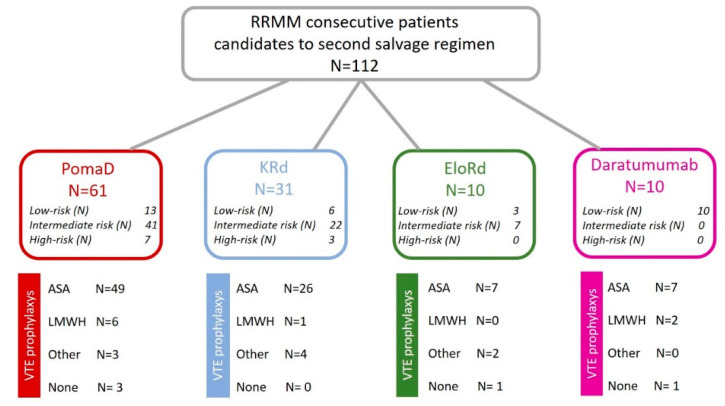
Regimens used as third-line of treatment in RRMM patients evaluated in this single-center retrospective study. The IMPEDE score was applied to distinguish patients in low, intermediate and high risk of VTE. For each treatment group, the VTE prophylaxis choice has been reported. Abbreviations: ASA: acetylsalicylic acid, LMWH: Low-molecular-weight heparin Dara: Daratumumab, EloRd: Elotuzumab, Lenalidomide, Desamethasone, KRd: Carfilzomib, Lenalidomide, Desamethasone, PomaD: Pomalidomide and Desamethasone.

We collected retrospectively data available for 112 patients, who received, as part of their second salvage regimen, respectively, 61 (54%) pomalidomide and dexamethasone (PomaD); 31 (28%) carfilzomib, lenalidomide and dexamethasone (KRd); 10 (9%) elotuzumab and dexamethasone (EloRd); and 10 (9%) daratumumab as single agent, as shown in Figure 1.

Demographic and clinical characteristics of RRMM patients included in the study are reported in Table 1. Median age was 69 years (range 45–87). Most patients were male (72 patients, 64%), 33% had a performance status (PS ECOG) higher than 2. Due to inclusion criteria of the study, all patients had received two previous lines of treatment including autologus or allogenic transplantation, and most of them had refractory disease to the last treatment.

Risk factors for thromboembolism in MM were categorized as patient-related and MM and therapy-related risk factors. Patient-related risk factors included obesity, inherited thrombophilic abnormalities, central venous catheter (CVC), comorbidities (such as infections, cardiac disease, chronic renal disease, immobilization), medications such as erythropoietin and recent surgical procedures (less than 3 months). MM-related risk factors were diagnosis per se as well as hyper- viscosity; therapy-related risk factors were treatment with high dose of dexamethasone, doxorubicin or multi-agent chemotherapy. As shown in Table 2, all patients had at least one individual risk-factor (91%), including co-morbidity (such as diabetes and COPD, chronic obstructive pulmonary disease, 75%) and anemia requiring supportive care with EPO (67%). According to NCCN, IMWG and EMN guidelines, 42 patients (38%) were classified as high-risk patients.

Since NCCN, EMN and IMWG guidelines fail to fully distinguish high and ultra-high-risk patients and are not addressed for the RRMM setting, we re-classified our patients according to the IMPEDE VTE score [51], which takes in account the following predictors of VTE: Immunomodulatory drugs; Body Mass Index > 25 Kg/m^2^; Pelvic, hip or femur fracture; Erythropoietin; Dexamethasone/Doxorubicin; Asian Ethnicity; VTE history; Tunneled catheter; Existing thromboprophylaxis. Each factor has different points ranging from 4 to −4 and this score is able to distinguish patients with a low (score ≤ 3), intermediate (score 4–7), and high (score ≥ 8) risk group. As shown in Figure 1, 32 (28%) patients were classified as low-risk, with a score ≤ 3 (most of them in the PomaD and Daratumumab group); 70 (63%) were classified as intermediate-risk, with a score 4–7 (most of them in PomaD and KRd group); and 10 (9%) were classified as high-risk, with a score ≥8 (most of them in the PomaD group).

### 3.2. VTE Thromboprophylaxis

As shown in Figure 1, all patients except 5 (4%) started VTE thromboprophylaxis at the same time of salvage regimen, including ASA in 89 (83%) patients, LMWH in 9 (8%) patients and 9 (8%) other kind of antiplatelet agents. Three patients started with LMWH and switched to ASA within the first six months of treatment due to patients’ refusal to practice daily subcutaneous administration; six patients, who started with ASA and one with lysine acetylsalicylate, switched to LMWH for increased VTE risk due to disease progression and increased number of individual risk factors. ASA was used in 49 (80%) patients in PomaD treatment, in 26 (84%) patients receiving KRd, in 7 (70%) patients in EloRd and in 7 (70%) patients in the Dara group. Most patients classified as high-risk according to IMWG classification refused LWMH due to low compliance; thus, ASA was prescribed. Regular and continued monitoring of hemogram for platelet counts was performed and none of patients included on the study developed thrombocytopenia requiring thromboprophylaxis reduction/interruption.

### 3.3. Description of Recorded VTEs

**Figure 2:**
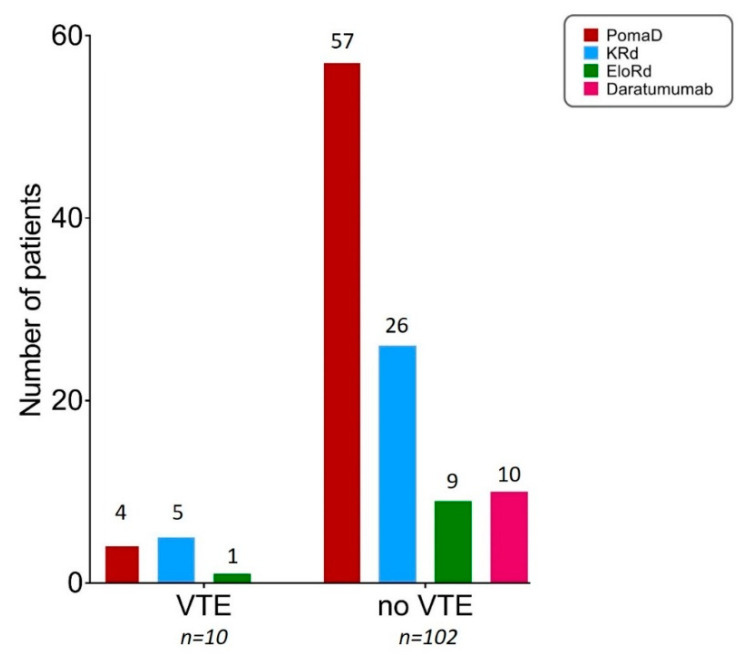
Distribution of VTE among RRMM patients treated with novel agents as part of their second salvage regimen.

As shown in Figure 2, in all, 10 (9%) patients treated with new agents developed VTE, including 7 uncomplicated DVT and 3 pulmonary embolism (PE). As shown in Table 2, no patients had therapy-related risk factors, including high dose dexamethasone defined as more than 480 mg/month [48]. All patients in the KRd and Daratumumab groups had at least one individual risk factors. Among MM-related risk factors we included hyperviscosity defined as IgG >15 g/dL, IgA >10 g/dL and for IgM >3 g/dL [48], present in five patients: two treated with pomalidomide and thre with KRd. Detailed analysis of both individual and MM-related risk factors for each treatment group is reported in Table 3. 3/10 patients had previous history of chronic venous disease (C1–C2 grade according to the CEAP criteria [52,53,54]), and no correlation was found between the CEAP score and VTE/PE severity.

Among PomaD treated patients who developed VTE, one patient had two individual risk factors (obesity and diabetes), whereas three patients had three or more individual- and MM-related risk factors. Despite the high-risk of developing VTE at the start of the salvage regimen, all of them refused LMWH prophylaxis and were receiving 100 mg ASA once daily at time of VTE development. DVT of lower extremities occurred in two patients within the first 2 months of treatment when the clinical anti-MM response was not yet achieved, and in two patients after 8 months of treatment, when a measurable response was documented. One patient, older than 75 years, affected by chronic obstructive pulmonary disease (COPD) and supported with EPO, developed PE with shortness of breath, chest pain, cough, requiring hospitalization, after 10 months of treatment, with a documented partial response. PE diagnosis was confirmed by computed tomography pulmonary angiography. At discharge, anti-MM treatment was discontinued. In all patients, pomalidomide was interrupted and three patients started a further line of salvage regimen based on daratumumab.

One patient in the KRd group who developed VTE had one individual risk factor (VTE history) while the remaining four had three or more individual risk factors and no MM-related risk factors. Despite the high-risk to develop VTE at start of salvage regimen, all of them refused LMWH prophylaxis and were receiving 100 mg ASA once daily at time of VTE development (*N* = 3) or clopidogrel (*N* = 2).

Two patients developed distal DVT within first two months from treatment start, and one after two years from treatment start, in the presence of a tunnel line. DVTs were treated with LWMH for 3 months, with recanalization of the occluded vessel, and lenalidomide was discontinued. Two patients experienced PE with shortness of breath, chest pain, cough: in both cases, diagnosis was confirmed by computed tomography pulmonary angiography. Compression ultrasonography of the lower limb revealed proximal DVT in 1 of them. PE events were at low risk because of absence of hemodynamic compromise, so they were both treated successfully with oral anticoagulant therapy for indefinite time and the anti-MM treatment switched to a further daratumumab-based regimen.

The only patient in the EloRd group who developed VTE had two individual risk factors (COPD, EPO), in absence of MM-related risk-factors (in very good partial remission), under ASA prophylaxis.

In the Daratumumab group, no VTEs were recorded.

According to the IMPEDE VTE score, none of the low-risk patients (*N* = 32) of our series had VTE. Among 70 intermediate-risk patients, 8 (11%) had VTE, including 4 in the PomaD group, 1 in the EloRd group and 3 in the KRd group. Among 10 high-risk patients, 2 (20%) had VTE, all in the KRd group.

## 4. Discussion

This is a retrospective unicentric study on real life actions for preventing and managing thromboembolic events in RRMM patients treated with novel drugs, outside of controlled clinical trials. We found that in RRMM patients, the incidence of VTEs is around 10%, despite the use of thromboprophylaxis (or anti-thrombotic treatment). Despite the study limitations, e.g., the retrospective nature of a real-life survey in a single center, the limited number of patients and events in each cohort, our observations have clinical implications.

Currently, in the absence of risk factors for thrombosis, prophylaxis with ASA (100 mg) is recommended for patients with MM who start IMiDs therapy and classified as low risk. Full dose anticoagulants (low molecular weight heparin or full-dose warfarin) are instead recommended for those at higher risk [38,39]. Our study included patients with myeloma who had a different risk of VTE, because of the presence of individual factors, such as obesity, recent surgery, presence of comorbidities, a history of VTE or treatment with erythropoietin [37]. Therefore, in our series, 38% of patients were classified as high risk, but only 8% were treated with LMWH, according to IMWG recommendation. This discrepancy can be attributed both to the will of patients who preferred oral therapy over daily subcutaneous administration, and to a widespread attitude of doctors who rely above all on a clinical evaluation of the individual patient, thus demonstrating a poor adherence to IMWG guidelines which were actually proposed without having been validated.

The poor concordance with IMWG guidelines regarding ASA or LMWH use for VTE prophylaxis in MM patients has been well documented in other studies dealing with “real life” experiences [48]. A retrospective French study conducted on 236 patients treated with an IMiDs-based regimen demonstrated that only 43% high-risk and 34% low-risk patients received a thrombo-prophylaxis [19]. Another study evaluated 80 MM cases with treatment-related VTE with 85% of them classified as high risk, but only 19% had received an appropriate prophylaxis according to IMWG [50]. The failure to adhere to the recommendations of the guidelines was also recorded in the context of controlled clinical studies: the English study Myeloma XI included treatment with IMiDs at diagnosis, an assessment of the thrombotic risk in accordance with the IMWG guidelines and consequently a prophylaxis with LMWH for high risk patients and with ASA for low risk patients. Despite these recommendations, almost 20% of patients did not receive any prophylaxis and 23% of patients classified as high risk underwent prophylaxis with ASA rather than LMWH [50]. A recent review has confirmed that ASA was the most frequent prophylaxis in patients treated with lenalidomide, no matter the individual risk assessment [44] and the most recent guidelines from NICE (National Institute for Health and Care Excellence) suggest that in patients with myeloma who are receiving treatment with thalidomide, lenalidomide or pomalidomide and steroids, it is possible to choose either aspirin or prophylactic dose of LMWH (NICE 2018 https://www.nice.org.uk/guidance/ng89). Indeed, many patients are reluctant to accept prophylaxis with LMWH, due to the discomfort of repeated and prolonged subcutaneous administration. Nonetheless, in our series few patients experienced VTE, even though they were under prophylaxis with ASA (100 mg), while some patients with three or more risk factors, either with PomaD or KRd, have not experienced thrombosis, thus confirming the inability of IMWG classification to well define the risk categories.

One of the reasons for the poor adherence of physicians to the IMWG indications could be related to the fact that the definition of the risk of developing a VTE remains too vague and the different parameters used to define the risk are equally considered, while they have probably a different strength as risk factors [48,49]. For example, the use of erythropoietin is considered a risk factor in the same way as others, but it must be taken into account that erythropoietin increases the risk of developing VTE especially when it determines a significant increase in hematocrit and hemoglobin (Hb) [19,55]. In our patients treated with erythropoietin, the Hb value has always been kept lower than 12 g/dL and therefore this treatment should not have increased the thrombotic risk even if this parameter was considered in the definition of the risk class, according to the indications of the IMWG [22]. Instead, a previous history of VTE seems to have a significant role in terms of defining the risk class [48]. It should be pointed out that a previous history of arterial or venous thromboembolism was an exclusion criteria for the two studies [36] comparing the three different approaches (ASA, LMWH and warfarin) as possible prophylaxis in IMiDs treated patients, that formed the basis for the formulation of the IMGW guidelines. Indeed, new studies have indicated that a previous VTE is the strongest indicator of the probability of developing VTE [51] and, in fact, one of our patients who developed VTE during EloRd had actually a history of VTE as the only risk factor; in these patients, ASA might be not enough to provide an adequate thrombo-prophylaxis [51].

All the most recent literature recognizes the need to give a different weight to the different risk factors. In this perspective, a new proposed score, the IMPEDE VTE score [51], that assigns different points to different risk factors, has been recently proposed. It has the merit of dividing patients in three categories with different risk of developing VTE. We found that this score in our series worked much better than the classification proposed by the IMWG, better defining patients at VTE risk. In fact, none of the patients classified as low risk developed VTE vs. 11% of intermediate risk patients and 20% of high-risk patients. Another prognostic model, the SAVED score, for the development of thrombosis has been recently proposed [52]. This model is also based on a different weight of different variables and it can identify two categories of patients at different risk of developing VTE. All these models are based on retrospective evaluations and have the major flaw that refer to now obsolete and no longer used anti-MM therapy schemes. Therefore, new prospective studies are needed that also consider not only the new therapeutic strategies in MM, but also the new anticoagulant drugs that are increasingly used both for therapeutic and prophylactic purposes.

Several studies have in fact shown a promising efficacy of the new oral anticoagulants (DOACs) in preventing thrombosis in cancer patients, including MM [40,49,51,55]. Recent studies also showed that apixaban, especially at low dose, was safe and effective for thromboprophylaxis in relatively small series of MM patients receiving an IMiD-containing regimen as first line treatment or for relapsed disease [53]. Therefore, although further studies are needed for evaluating the role of DOACs in VTE prophylaxis in MM patients and possible drug interactions between DOACs and specific myeloma treatment, including supportive medications, these drugs seem to be at least as effective as LMWH and are an attractive alternative for thromboprophylaxis in MM since they do not require monitoring and spare patents from daily injection [48]. However, DOACs are expensive and in many countries they are not licensed for prophylaxis. Therefore, a more accurate risk stratification of VTE risk should be pursued not only for clinical benefits, but also for economic reasons [48]. However, it remains always difficult to accurately define the real risk for each individual patient. It is possible that in the future, some laboratory investigations, especially those that evaluate the global hemostasis such as the thrombin generation [10] may better identify high-risk patients. Nowadays, a careful clinical and anamnestic evaluation of the patient and a physician awareness of the thrombotic risk remain the only reliable tools to define the risk class.

Despite the limitations of this study, given by its real-life nature, as a retrospective study with single-center data collection, there are some interesting emerging points. First, we confirmed the lower incidence of VTEs in RRMM patients treated with monoclonal antibodies. This could be due to the baseline characteristics of patients, with fewer risk factors in Elo and Dara groups or the limited sample size. However, our findings in the community setting confirm what previously reported in clinical trials [48]. Even if daratumumab as single agent is lesser used than in the past, there is an increasing number of patients that will receive maintenance with daratumumab single agent [56] and that could benefit from our findings. In general, the paradigm of long-term treatment with elotuzumab and daratumumab is increasingly well-established across treatment settings, potentially resulting in further improvements in patient outcomes, and highlighting key clinical issues that will need to be addressed in order to provide optimal benefit, including the VTE prophylaxis and for how long it should be required.

Second, in half of the patients, VTE occurred during the first months of treatment, confirming the role of an active disease in the pathogenesis of VTE. This had clinical consequences only in two patients who discontinued anti-MM treatment, while others switched to a different regimen, omitting IMiDs (e.g., switch to daratumumab), suggesting that evaluation of VTE risk should be dynamic, and repeated at each cycle of treatment to personalize the prophylaxis options. To this end, a survey is ongoing in Italian centers to establish which options should be offered for VTE prophylaxis and to sensitize hematologists to this issue in the community setting. In future, the IMPEDE score should be validated in RRMM patients undergoing continuous therapy as part of second and third-line salvage regimens. Among criteria for choosing the third-line regimen to offer, it could be worthwhile to include the VTE risk and to address daratumumab-based regimens to those high-risk patients who refuse LMWH prophylaxis, or include DOACs as an alternative VTE prophylaxis.

## 5. Conclusions

This is the first report about the incidence of VTE in a real-life cohort of RRMM patients treated with novel agents, including monoclonal antibodies. Given the changes in treatment options for MM patients, with more and more patients treated in second and third line, larger prospective series are required to develop a dynamic score that could summarize the individual-, MM-related and treatment-related risk and to recommend the adequate thromboprophylaxis.

## Figures and Tables

**Table 1:** Demographic and clinical characteristics of MM patients included in the study.

	Poma D	KRd	Daratumumab	EloRd
	*N* = 61 (100%)	*N* = 31 (100%)	*N* = 10 (100%)	*N* = 10 (100%)
**Median age (range)**	71 (45–87)	66 (50–78)	66 (52–74)	61 (48–72)
**Males, n (%)**	39 (64)	22(71)	6(60)	5 (50)
**ECOG performance status, n (%)**				
0–2	30 (49)	26 (84)	9 (90)	10 (100)
>2	31 (51)	5 (16)	1 (10)	0 (0)
**International Staging System stage, n (%)**				
I	5 (9)	10 (32)	8 (80)	8 (80)
II	22 (36)	5 (16)	2 (20)	1 (10)
III	34 (55)	16 (52)	0 (0)	1 (10)
**Cytogenetics risk (FISH) available at baseline**	39 (64)	23 (74)	9 (90)	8 (80)
**High-risk**	13 (21)	11 (35)	3 (30)	3 (30)
**Standard-risk**	26 (43)	12 (39)	6 (60)	5 (50)
**Glomerular filtration rate (GFR, mL/hour)**				
>60 mL/hour	54 (89)	13 (43)	2 (20)	6 (60)
30–60 mL/hour	5 (10)	5 (17)	6 (60)	4 (40)
<30 mL/hour	2 (1)	3 (10)	2 (20)	0 (0)
**Median haemoglobin (range), g/dL**	9.3 (7.1–12.2)	9.5 (7.8–14.6)	10.1 (8.1–14.6)	10.2 (7.2–11.6)
Median platelet count (range), *10^3^/mmc	145 (30–316)	130 (20–560)	136 (25–198)	156 (70–215)
**Median monoclonal component (range), g/dL**	2.3 (0.3–3.2)	1.6 (0.2–2.1)	0.9 (0.1–3.1)	0.5 (0.1–2.0)
**Previous treatments**				
Autologous transplantation	12 (20)	15 (48)	5 (50)	3 (30)
Allogeneic transplantation	4 (6)	0 (0)	2 (20)	0 (0)
Continuous lenalidomide	61 (100)	7 (23)	2 (20)	0 (0)
**Disease status**				
Refractory disease	36 (59)	17 (55)	5 (50)	8 (80)
Aggressive relapse	8 (13)	5 (16)	0 (0)	2 (20)
Biochemical relapse	17 (28)	9 (29)	5 (50)	0 (0)

ECOG: Eastern Cooperative Oncology Group, MM: Multiple myeloma, PomaD: Pomalidomide and Dexamethasone; KRd: Carfilzomib, Lenalidomide, Dexamethasone; EloRd: Elotuzumab and Lenalidomide.

**Table 2:** Data on individual, MM-related and anti-MM therapy-related VTE risk factors.

Risk Factors	PomaD *N* = 61 (100%)	KRd *N* = 31 (100%)	EloRd *N* = 10 (100%)	Daratumumab *N* = 10 (100%)	Total *N* = 112 (100%)
**Individual**	**55 (90)**	**31 (100)**	**6 (60)**	**10 (100)**	**102 (91)**
Obesity (BMI > 30 kg/m^2^)	22 (35)	13 (42)	1 (10)	1(10)	37(33)
Previous VTE	1 (2)	2 (6)	0 (0)	0 (0)	3 (3)
CVC or PM	1 (2)	4 (13)	0 (0)	1 (10)	6 (7)
Associated comorbidities	43 (70)	30 (97)	4 (40)	7 (70)	84 (75)
Surgical procedures	7 (11)	7 (23)	1 (10)	3 (30)	18 (16)
Erythropoietin	50 (82)	19 (61)	6 (60)	6(60)	75 (67)
**MM-related**	**47 (78)**	**17 (55)**	**6 (60)**	**6 (60)**	**78 (70)**
Hyperviscosity	2 (3)	3(10)	0 (0)	0 (0)	5 (4)
Active uncontrolled disease	45 (74)	17 (55)	6 (60)	6 (60)	74 (66)

MM: Multiple myeloma, PomaD: Pomalidomide and Dexamethasone; KRd: Carfilzomib, Lenalidomide, Dexamethasone; EloRd: Elotuzumab and Lenalidomide; BMI: body mass index; VTE: venous thromboembolism; CVC: central venous catheter; PM: pace maker.

**Table 3:** Analysis of risk factors for VTE among RRMM patients treated with novel agents.

Treatment Group	Number of Subjects	Number of Individual and MM-Related Risk Factors		
		≤1	2	3 or More
PomaD	61 (100)	14 (23)	19 (31)	28 (46)
No VTE	57 (93)	14 (23)	18 (29)	25 (41)
VTE	4 * (7)	0 (0)	1 (2)	3 (5)
KRd	31 (100)	5 (16)	8 (26)	18 (58)
No VTE	26 (84)	4 (13)	8 (26)	14 (45)
VTE	5 ** (16)	1 (3)	0 (0)	4 (13)
EloRd	10 (100)	4 (40)	4 (40)	2 (20)
No VTE	9 (90)	4 (40)	3 (30)	2 (20)
VTE	1 *** (10)	0 (0)	1 (10)	0 (0)
Daratumumab				
No VTE	10 (100)	4 (40)	5 (50)	1 (10)
VTE	0 (0)	0 (0)	0 (0)	0 (0)

* Patient #1 had 4 risk factors (obesity, diabetes, EPO and hyperviscosity); patient # 2 had 3 risk factors (obesity, diabetes, EPO); patient #3 had 3 risk factors (history of VTE, chronic obstructive pulmonary disease, EPO, tunneled catheter); patient #4 had 3 risk factors (obesity, EPO, history of VTE). ** Patient #1 had 3 risk factors (obesity, tunneled catheter, EPO); patient #2 had one risk factor (VTE history), patient #3 and #4 had 4 risk factors (obesity, tunneled catheter, EPO, diabetes); patient #5 had 3 risk factors (chronic obstructive pulmonary disease, EPO, tunneled catheter). *** Patient #1 had 2 risk factors (chronic obstructive pulmonary disease, EPO). Abbreviations: KRd: Carfilzomib, Lenalidomide, Desamethasone, MM: Multiple Myeloma, PomaD: Pomalidomide, VTE: venous thromboembolism, EPO: erythropoietin.

## Appendix A

Treatment with PomaD consisted of 28/35-day cycles of pomalidomide given 4 mg daily per os on days 1–21 of each 28-day cycle and dexamethasone 40 mg weekly (for <75 years patients) and 20 mg weekly (for ≥75 years patients) until progression. Treatment with KRd consisted of 28-day cycles of KRd regimen consisted of 28-days cycles with Carfilzomib administered intravenously for two consecutive days a week, for three weeks. Initial dose was 20 mg/m^2^ (maximum dose 40 mg) on days 1–2 of first cycle, increased to 27 mg/m^2^ (maximum dose 60 mg) at day 8 of first cycle, if well tolerated. Carfilzomib administration was interrupted at days 8–9 from 13th cycle onwards. Lenalidomide was usually administered 25 mg per os on days 1–21 of each 28-day cycle and dexamethasone 40 mg weekly (for <75 years patients) and 20 mg weekly (for ≥75 years patients) until progression.

Dara regimen consisted of 4-weeks cycles of Daratumumab given 16 mg/kg intravenous (iv) infusion weekly for the first two cycles, followed by intravenous administration every two weeks on weeks 9–24 and finally once every four weeks from week 25 until progression.

EloRd regimen consisted of 28-days cycles of Elotuzumab intravenously administered at dose of 10 mg/kg weekly for the first two cycles and then every two weeks, Lenalidomide given 25 mg per os on days 1–21 of each 28-days cycle and, dexamethasone administered per os at a dose of 40 mg during the week without elotuzumab and intravenously at a dose of 8 mg plus 28 mg orally on the day of elotuzumab administration until progression.
